# Evaluating customer perspectives on omnichannel shopping satisfaction in the fashion retail sector

**DOI:** 10.1016/j.heliyon.2024.e36027

**Published:** 2024-08-09

**Authors:** Bilal Khalid

**Affiliations:** KMITL Business School, King Mongkut's Institute of Technology Ladkrabang, Bangkok, Thailand

**Keywords:** Omnichannel, Customers perspective, Integrated promotions, Integrated customer service, And integrated transactions

## Abstract

The effective implementation of omnichannel commerce can fundamentally alter how consumers shop online. This study attempted to understand Thai consumers' omnichannel fashion retail purchasing activities. The objectives of the study were to investigate the determinants shaping omnichannel customer experiences within the fashion retail industry and to examine the impact of omnichannel customer experiences on customer satisfaction within the Thai retail industry. The research utilized the Unified Theory of Acceptance and Use of Technology (UTAUT) model to analyze the effects of omnichannels on purchasing behaviors and levels of satisfaction of consumers. The study employed a survey research design, applying simple random sampling to select 509 respondents with omnichannel shopping experience in the clothing and fashion. The respondent data was analyzed using structural equation modeling utilizing the Amos software version 24. Analyzing the results revealed a significant correlation between omnichannel shopping and customer satisfaction in fashion retail shopping. Perceived ease of use, perceived enjoyment, integrated promotions, integrated customer service, and integrated transactions were all found to influence omnichannel experiences favorably. The findings suggest that fashion retailers prioritize customer satisfaction by enhancing their omnichannel experiences through better coordination and synchronization of their different customer service channels.

## Highlights

• Studied omnichannel customer experiences in the Thai fashion retail sector, aiming to understand determinants shaping these experiences and their impact on customer satisfaction.
• Utilized the UTAUT model for analysis, surveying 509 respondents with omnichannel shopping experience for fashion retail.
• Analyzed data through structural equation modeling with Amos software, revealing a significant correlation between omnichannel shopping and customer satisfaction.
• Identified influencing factors such as perceived ease of use, perceived enjoyment, integrated promotions, integrated customer service, andintegrated transactions in shaping positive omnichannel experiences.
• Suggested fashion retailers prioritize enhancing omnichannel experiences by better coordinating their various customer service channels to improve customer satisfaction.

## 1 Introduction

Omnichannel marketing is an advanced method of e-commerce aimed at offering customers an integrated and uniform experience across multiple channels as they explore their shopping options. Being specific, physical storefronts and online platforms are all constituents of omnichannel. Mobile and web applications, websites, and social media platforms are all part of the online channels available to merchants [1,2]. Merchants aim to create a reliable and interconnected platform to enhance the brand and shopping experience for customers. This allows customers to easily connect with the retailer through different touchpoints and move effortlessly between channels. Thus, the conversation could begin with an inquiry on social media, and the customer is directed to the specific webpage where they can find the requested items. The customer can order online and have the item delivered to his physical address or go to the storefront to confirm that what is being ordered is available in the shape and form it appears online. The client can pay physically or use any online channel available. Statista estimates that by 2026, online retail sales will have risen from $5.2 trillion in 2021 to over $8.1 trillion, representing a growth rate of 56 % forthcoming [3]. The change is attributed to the rising digital technology penetration level and the evolving consumer preferences toward ease and personalization of user experience.

Scholars are of the view that applying digital technology to shopping has greatly disrupted the retail sector for most products [4]. Retailers are now compelled to adopt an omnichannel approach to remain competitive and meet evolving customer needs. Omnichannel, as defined by Sorkun et al. [5] is a multi-channel sales strategy that unifies the buying experience for consumers across traditional physical stores, smartphone apps, and social platforms. Merritt and Zhao [6] assert that omnichannel provides customers with a consistent and integrated experience regardless of their shop. A true omnichannel experience means customers effortlessly transition from one channel to the next without starting the buying process from scratch. Due to the progress in technological innovation, companies now have the capability to engage in novel and imaginative ways with their customers. For example, social media networks such as Instagram and Facebook enable retailers to reach a wider audience and actively interact with customers in real time, allowing them to showcase their products effectively [7,8].

Also, it has been acknowledged that mobile device use has significantly impacted the retail sector. They affirm the increasing use of smart phones and internet-enabled hand-held devices to browse and shop for products by consumers, thus enabling the development of mobile apps and responsive websites that enable customers to shop online, eliminating geographical barriers to retail shopping [9,10]. Cotarelo et al. [11] state that using AU/AI/VR/ML has further transformed the customer experience. The COVID-19 pandemic has accelerated technology adoption, with retailers now focusing on enhancing their online presence and offering contactless delivery options [[12], [13], [14]].

The retail industry has adopted omnichannel to give consumers a smooth and comprehensive shopping experience, especially in the fashion retail sector, where merchants utilize omnichannel methods to boost customer engagement and sales [15]. Thaichon et al. [16] highlights how retailers are integrating their online and offline channels by offering in-store pickup options for online purchases and allowing customers to return products in-store or online. According to a report by McKinsey and Company, the omnichannel sales growth in the fashion retail sector is expected to outpace e-commerce sales growth in the next few years. Based on Statista's data for 2021, the Thailand fashion sector generated proceeds of $4.94B USD. Furthermore, the Consumer Market Outlook of Statista forecasts that this figure will increase to $7.21B USD by 2026 [17]. The integration of omnichannel strategies in the fashion retail sector has become essential to meet the growing customer demand for unified and personalized retail interactions.

Despite omnichannel shopping being increasingly prevalent globally, there is insufficient scholarship of this subject area in the Thai context, especially as applicable to fashion retailing sector. The challenge of understanding the factors influencing customer satisfaction in the omnichannel purchasing experience in the Thai fashion retail industry is addressed in this paper, as more research is required to understand how aspects like integrated services, perceived enjoyment, perceived value, and perceived ease of use affect consumer satisfaction. Although some studies have examined omnichannel retailing on its merit, the Thai market invariably has distinguishable attributes that may influence the outcome of an omnichannel-level marketing approach [[18], [19], [20]]. Therefore, there is a need to study customer perspectives regarding omnichannel shopping satisfaction in the fashion retail sector in Thailand. The investigation aims to thoroughly explore the various factors that significantly affect customers' satisfaction levels when engaging in omnichannel shopping within Thailand's fashion industry. By achieving these objectives, this study can contribute to understanding omnichannel retailing in the Thai context and provide insights for fashion retailers on improving their omnichannel strategy to enhance customer satisfaction and loyalty. The author has proposed the under listed objectives to guide the investigation of omnichannel customer satisfaction in fashion retail in Thailand, they include.

i. To investigate the determinants shaping omnichannel customer experiences within the fashion retail industry.
ii. To examine the impact of omnichannel customer experiences on customer satisfaction within the retail industry.

Theoretically, this research aims to extend the Unified Theory of Acceptance and Use of Technology (UTAUT) model of Venkatesh et al. [21] by integrating it with specific omnichannel aspects such as perceived ease of use, perceived enjoyment, perceived value, integrated promotions, integrated customer service, integrated transactions, and integrated order fulfillment. This integration is expected to provide a framework for understanding the determinants of customer satisfaction from an omnichannel standpoint, focusing on the fashion retail sector in Thailand; offering practical and relevant information for fashion shops aiming to enhance their omnichannel strategies, emphasizing the importance of perceived ease of use, perceived enjoyment, and perceived value in enhancing the customer experience, proposing a clear roadmap for retailers to improve these aspects through user-friendly interfaces, engaging content, and value-added services as identified in the literature [16,[22], [23], [24]]. Key determinants addressed by the study focuses on the unique attributes of the Thai fashion retail market. The study emphasizes the need for integrated customer service and integrated transactions in promoting customer satisfaction [25] and recommends that merchants concentrate on providing smooth service and effective transaction procedures through all channels [26].

By addressing these core areas, fashion retailers in Thailand and similar markets can enhance customer satisfaction and loyalty, ultimately driving sales and competitive advantage [27]. Prior studies explored the benefits and challenges of omnichannel strategies, highlighting the importance of a seamless customer experience across multiple channels; providing generic information that are lacking in the specific characteristics of Thailand's fashion industry [1,2,16]. This study builds on these findings by providing empirical evidence from the Thai fashion retail sector, thus filling a geographical gap in the literature. From a theoretical perspective, the research by Gao et al. [26] on consumer behavior in omnichannel contexts and Quach et al. [28] on service delivery integration informs the theoretical underpinnings, and validates existing knowledge while offering new perspectives specific to the Thai market, improving the understanding of omnichannel retailing's impact on customer satisfaction by comparing the theoretical framework with seminal works.

## **2** Literature review

### 2.1 Customer satisfaction in the context of omnichannel retailing

Recent advancements in omnichannel retailing have significantly transformed customer satisfaction paradigms, driven by the seamless integration of various channels, including physical stores, online platforms, mobile applications, and social media [[29], [30], [31], [32], [33]]. The state-of-the-art research in this field demonstrates the importance of delivering a cohesive and personalized customer experience across all channels of interaction with customers [[34], [35], [36]]. Some retailers consider boosting customer satisfaction by providing a consistent purchasing experience across many channels [26,[37], [38], [39], [40]]. In a systematic analysis, Furquim et al. [41] identified three phases of the omnichannel buying process, each presenting different opportunities and challenges for merchants to engage with customers. Cotarelo et al. [11] argue that customer satisfaction and fidelity are strongly correlated with omnichannel incorporation intensity and the perceived value of customers' purchases. Regarding omnichannel commerce, Huang and Shi [42] analyzed the problem of where to put the front distribution center and provide a mathematical model to maximize the site choice based on criteria including customer demand, delivery costs, and inventory management. The study suggests that companies use a data-driven approach to optimize their supply chains and better serve their customers with timely and accurate deliveries. The research conducted by Shukla and Tandon [43] alludes that merchants must prioritize delivering unified and personalized encounters for shoppers on all platforms. This approach is critical in boosting client engagement while fostering fidelity.

Key findings reveal that omnichannel strategies enhance customer satisfaction by providing convenience, consistency, and real-time information, fostering loyalty and repeat purchases [[44], [45], [46]]. Studies highlight that personalized communication and tailored offers, facilitated by data analytics and artificial intelligence, are crucial in meeting customer expectations [34,35]. Integrating augmented reality (AR) and virtual reality (VR) has enriched the shopping experience by offering immersive product interactions; while synchronizing inventory and customer data across channels ensures customers access accurate product availability and personalized recommendations [34,35]. Jasrotia [47] highlights the importance of enhancing customers' shopping habits in the modern retailing age amidst benefits derived from interactive marketing made possible by technological advances [48]. The research recommends a customer-centric approach and the use of technologies like AI, big data, and machine learning so that organizations may provide their customers with unique and engaging experiences. Mahadevan and Joshi's [49] study on the analysis of citations and social networks of omnichannel retailing proposes that businesses prioritize providing uniform and unified retail encounter throughout available platforms and use technologies. To reduce their negative impact on the environment and boost consumer satisfaction, firms should establish a sustainable strategy and apply eco-friendly methods like green logistics, renewable energy, and the circular economy [[50], [51], [52]]. Cattapan and Pongsakornrungsilp [53] investigated how Millennials' views on omnichannel shopping affect their behavior in fashion businesses. They argue that stores must put Millennials' needs first by providing a uniform and individualized multi-channel retailing experience while using social media and influencer marketing to connect with them and gain their patronage. Despite these advancements, challenges such as data privacy concerns and the complexity of managing omnichannels persist [16,54]. Research indicates that addressing these issues through vigorous security measures and frameworks using streamlined operations is vital for maintaining customer trust and satisfaction. Notably, the state-of-the-art approaches in omnichannel retailing emphasize a customer-centric model, leveraging technological innovations to create a seamless and satisfying shopping journey [16,54,55].

Although there have been significant gains in omnichannel retailing and its ability to enhance consumer satisfaction, a considerable theoretical gap exists in understanding the precise mechanisms through which these integrated experiences influence customer loyalty and long-term engagement in Thailand; current research predominantly focuses on the immediate benefits of omnichannel strategies, such as convenience and consistency, without delving deeply into the underlying psychological and behavioral processes that drive sustained customer satisfaction and loyalty [16,56,57]. Moreover, while the role of technologies like data analytics, AI, AR, and VR in enhancing customer experiences is well-documented [22,58], there needs to be more comprehensive theoretical frameworks that explain how these technologies interact with traditional retail elements to create a holistic customer experience. Existing studies tend to isolate technological impacts from other critical factors such as emotional engagement, perceived value, and trust, leading to a fragmented understanding of the omnichannel customer journey. This theoretical gap suggests the need for an integrated model incorporating technological and non-technological factors, exploring how they collectively contribute to a seamless and satisfying customer experience. Thus, further theoretical discourse will illustrate the long-term effects of omnichannel strategies on customer behavior, particularly in terms of loyalty, integrated promotion, and integrated customer service. Addressing this gap can provide a better foundation for retailers to develop effective omnichannel strategies that attract and retain customers over time.

### 2.2 Empirical literature and hypotheses formation

#### 2.2.1 UTAUT model in technology adoption

The Unified Theory of Acceptance and Use of Technology (UTAUT) model is a theoretical framework Venkatesh et al. [59] developed to understand and explain individuals' acceptance and usage of technology [37,60,61]. The UTAUT model is a valuable framework that considers numerous factors influencing people's choices to adopt and utilize technology. It provides valuable perspectives on how customers accept and use technology and is extensively applied in this domain. The UTAUT model was selected for this study due to the expansive nature of its framework, which integrates critical determinants influencing technology acceptance and usage [61,62]. UTAUT's ability to incorporate variables such as perceived ease of use, perceived enjoyment, and perceived value aligns well with the study's focus on understanding customer satisfaction in omnichannel retail [63,64]. Moreover, UTAUT has a strong theoretical foundation and has been proven applicable in various settings [65]. These points make the UTAUT model ideal for studying the complex interactions between technological and human factors in omnichannel fashion retailing in Thailand. In omnichannel shopping, Chaudhary et al. [66] state that the UTAUT framework offers valuable insights into customers' perspectives on the ease of use, perceived enjoyment, and perceived value of the omnichannel shopping experience. In this literature review, we explored empirical studies that have investigated the UTAUT model and its relationship with customers' perspectives on omnichannel shopping satisfaction in the fashion retailer sector in Thailand.

#### 2.2.2 Perceived ease of use

Numerous studies indicate an association linking the perceived ease of use and satisfaction with omnichannel shopping [11,[67], [68], [69]]. For instance, Cattapan and Pongsakornrungsilp [53] found that the perceived ease of use of omnichannel shopping was positively correlated with Millennials' purchase intention for fashion retailers. Similarly, Chaudhary et al. [66] discovered that perceived ease of use influenced Indian millennials' desire to participate in omnichannel buying for fashion items considerably. These findings suggest that retailers must provide a user-friendly, easy-to-use omnichannel platform to enrich customer satisfaction. To investigate these in the Thai fashion retail industry, I proposed the following hypothesis.

Hypothesis 1 Perceived ease of use significantly influences omnichannel customer experience in the fashion retailer sector.

#### 2.2.3 Perceived enjoyment

Recent publications have hinted how perceived enjoyment is critical to shaping customers' attitudes toward omnichannel shopping [11,[70], [71], [72], [73], [74], [75]]. Cotarelo et al. [11] found that perceived enjoyment significantly predicted customer satisfaction and loyalty in omnichannel shopping. Likewise, the work of Furquim et al. [41] revealed that the concept of pleasure is crucial in delineating the different phases highlighting consumer purchasing journey in the omnichannel context. These findings emphasize the significance for retailers to concentrate on crafting a positive omnichannel shopping experience to boost customer satisfaction [76]. The aforementioned hypothesis is put forward.

Hypothesis 2 Perceived enjoyment significantly affects omnichannel customer experience.

#### 2.2.4 Perceived value

Perceived value is critical in shaping customers' attitudes towards omnichannel shopping, as highlighted in recent studies [49,50,72,73,77]. Cai and Lo [78] demonstrated a strong link between perceived value and consumers' willingness to adopt omnichannel platforms. This implies that customers' perception of the value they receive from various channels significantly influences their decision to embrace omnichannel shopping. Sousa et al. [51], in linking the variable to the environment, opined that perceived value is integral and paramount when the objective is sustainability; their exploratory study was conducted on the Brazilian omnichannel retail industry. Their findings emphasized the need to provide customers with perceived value across multi-channels to support long-term success and sustainability. These research findings emphasize the significance of retailers prioritizing delivering high perceived value in the omnichannel shopping experience. By offering compelling benefits, personalized experiences, competitive pricing, convenient services, and seamless integration across channels, retailers can enhance customers' perceived value and increase their satisfaction with the omnichannel shopping journey [72,79]. Hypothesis three focuses on perceived value which allows retailers to meet customer expectations, build loyalty, and gain a competitive edge in the evolving landscape of omnichannel retail.

Hypothesis 3 Perceived value significantly influences Omnichannel customer experience.

#### 2.2.5 Omni-channel predictors

Omnichannel retailing has grown in importance in Thailand's fashion retailer industry since it delivers consumers a seamless shopping experience across many platforms. The section explores the impact of integrated promotion, integrated customer service, integrated transactions, integrated order fulfillment, and omnichannel customer experience on customers' satisfaction with omnichannel shopping in the fashion retailer sector in Thailand.

#### 2.2.6 Integrated promotion

Integrated promotion seamlessly integrates various promotional tools and communication channels, delivering a cohesive and consistent message to customers across all available platforms [[80], [81], [82]]. Integrated promotion, under the framework of omni-channel promotion, pertains to the synchronized and unified strategy for promotional efforts spanning multiple platforms employed by an organization. It entails coordinating promotional communication, incentives, and strategies between channels across digital and physical locations to deliver cohesive and consistent customer service that will lead to transactions and order fulfillment [57]. Cattapan and Pongsakornrungsilp [53] demonstrated that integrated promotion positively impacts millennials' purchase intention regarding fashion retailers. This suggests that when promotional efforts are strategically coordinated and unified across different channels, they can effectively influence millennials' purchase decisions. Huang and Shi [42] conducted a study that revealed the significant impact of integrated promotional strategies on customers' omnichannel shopping behavior. Their findings highlighted the importance of a well-coordinated and consistent promotional approach in shaping customers' preferences and behaviors across multiple channels, and lead to the arguments of the fourth hypothesis.

Hypothesis 4 Integrated promotion significantly influences omnichannel customer experience.

#### 2.2.7 Integrated customer service

In maintaining all-round business communication, an integrated customer service is of paramount importance in ensuring a uniform and uninterrupted support experience for customers through various communication channels [83,84]. Cotarelo et al. [11] revealed that the intensity of omnichannel engagement and perceived shopping value are significant determinants of consumer happiness and loyalty, leading to more transactions between the business and its customers [1]. These findings suggest that by offering integrated customer service, fashion retailers can positively impact customers' satisfaction with omnichannel shopping in the Thai fashion retail sector. Following the discussions, we put forward the fifth research hypothesis.

Hypothesis 5 Integrated customer service significantly influences omnichannel customer experience.

#### 2.2.8 Integrated transactions

Integrated transactions in omnichannel shopping encompass integrating various payment options and channels to ensure customers a seamless and consistent payment experience [37,85,86]. Chen et al. [87] emphasized the significance ascribed to the quality of channel integration in influencing responses by customers' in omnichannel retail approach. This finding suggests that when different transaction channels are effectively integrated, it can positively impact customers' satisfaction with omnichannel shopping in the fashion retailer sector in Thailand. One way of doing this is to keep open the open of starting a transaction online and concluding in a physical store offline, or by starting transactions in a physical store and concluding online with payment and order fulfillment [26]. By integrating payment options and channels, fashion retailers can offer customers a convenient and flexible payment experience, regardless of the channel they choose to make purchases [88]. This integration may involve options like credit cards, mobile wallets, online payment platforms, and in-store payment methods, impeccably interconnected to facilitate secure transactions to ease the customers' minds. The discussion on integrated transactions led to proposing hypothesis six as follows.

Hypothesis 6 Integrated transactions significantly influence Omnichannel customer experience.

#### 2.2.9 Integrated order fulfillment

Integrating diverse fulfillment channels is crucial to providing customers with a streamlined and consistent order fulfillment experience [[89], [90], [91]]. Sousa et al. [51] emphasized that sustainability poses a significant challenge in omnichannel retailing. These findings suggest that integrated order fulfillment can positively impact customers' satisfaction with omnichannel shopping in the fashion retail sector. Businesses that promote their products should have good customer services available to ensure that transactions are seamless, and orders are promptly fulfilled to the satisfaction of customers. Fashion retailers can ensure that customers receive a unified and efficient order fulfillment process by integrating fulfillment channels, regardless of the channel through which they place their orders. This integration may involve connecting brick-and-mortar stores, warehouses, online platforms, and third-party logistics providers to facilitate inventory visibility, accurate order processing, efficient delivery, and hassle-free returns [92]. Considering the research findings, we hypothesize that integrated order fulfillment positively influences customers' satisfaction with omnichannel shopping.

Hypothesis 7 Integrated order fulfillment significantly influences omnichannel customer experience.

#### 2.2.10 Omnichannel customer experience

Thaichon et al. [16] emphasize how vital an omnichannel experience is in providing customers with a seamless and harmonious service on multi-platforms. This seamless and cohesive experience is crucial for customer satisfaction. Furquim et al. [41] accentuated the significance of the omnichannel customer experience in various stages of the consumer purchasing process. Their findings stressed the importance of delivering customers a seamless and personalized experience across all touchpoints. Furthermore, Jiang and Stylos [12] harped on the value of providing customers with a personalized, interactive experience across channels. They noted that customers are more likely to engage with retailers who offer such experiences, contributing to increased customer satisfaction and loyalty. This aligns with the evolving expectations of modern consumers who seek tailored and interactive interactions with brands, as identified by other scholars [26,93,94]. In addition, Cattapan and Pongsakornrungsilp [53] argue that omnichannel integration significantly influences the purchase intention of Millennials when it comes to fashion retailers. This further supports the notion that a well-integrated and cohesive omnichannel experience can positively impact customers' perceptions and behaviors. From the analyzed research, we present the subsequent hypothesis.

Hypothesis 8 Omnichannel customer experience significantly influences customers' satisfaction with omnichannel shopping.

Hypothesis 9a Omnichannel customer experience mediates the effect of perceived value on omnichannel customer satisfaction.

Hypothesis 9b Omnichannel customer experience mediates the effect of perceived enjoyment on omnichannel customer satisfaction.

Hypothesis 9c Omnichannel customer experience mediates the effect of perceived ease of use on omnichannel customer satisfaction.

Hypothesis 9d Omnichannel customer experience mediates the effect of integrated promotion on omnichannel customer satisfaction.

Hypothesis 9e Omnichannel customer experience mediates the effect of integrated customer service on omnichannel customer satisfaction.

Hypothesis 9f Omnichannel customer experience mediates the effect of integrated transactions on omnichannel customer satisfaction.

Hypothesis 9g Omnichannel customer experience mediates the effect of integrated order fulfillment on omnichannel customer satisfaction.

### 2.3 Conceptual framework

**Fig. 1:**
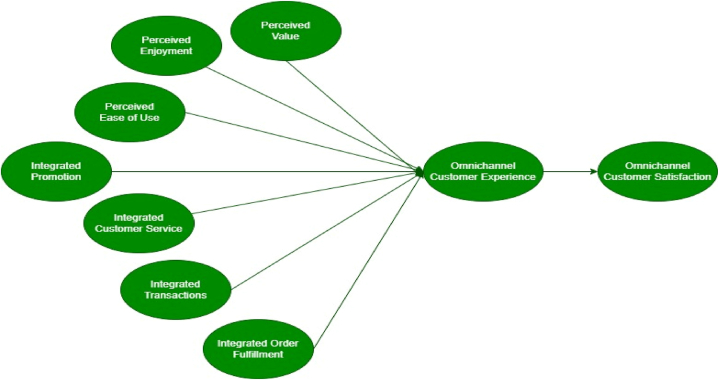
Adopted conceptual framework.

Developing a solid structure is essential for conducting a comprehensive study. This study established the conceptual framework by carefully reviewing relevant literature and formulating hypotheses. The framework encompasses a set of independent and dependent variables crucial in identifying variables influencing customer omnichannel experience and their satisfaction within the fashion retail sector. Drawing from the widely accepted UTAUT model, three independent variables were identified for inclusion in the framework. These variables under consideration include perceived enjoyment, perceived value and perceived ease of use, regarding its importance. Perceived enjoyment variables capture the level of pleasure and satisfaction experienced by customers during their interactions with the omnichannel platform. Perceived value is regarded in terms of the customer perception of accruable incentives and value in utilizing omnichannel services (see Fig. 1.).

In addition to the stated variables, four independent variables were included in the research framework to capture the specific aspects that bother on omnichannel predictors. These variables are integrated promotion, integrated transactions, integrated order fulfillment and integrated customer services. Interestingly, integrated promotion emphasizes the seamless integration of promotional activities across various channels to deliver a consistent message to customers. Integrated customer services concentrate on establishing a uniform and smooth support experience for customers on supported platforms. Integrated transactions involve incorporating different payment options and channels to ensure a seamless and convenient payment experience. Integrated order fulfillment aims to provide customers of the business with a streamlined and consistent order fulfillment procedure across all operation channels. The dependent variables in the conceptual framework are omnichannel customer experience and customer satisfaction. These variables serve as key indicators of the effectiveness and success of the omnichannel strategy implemented by fashion retailers. By investigating the links existing between the dependent and independent components, the research provides insights into the factors influencing customers' experiences and satisfaction utilizing omnichannel when shopping for fashion.

## 3 Research methods

This research focused on the experiences of shoppers using omnichannel in fashion retailing. The research focused on the respondents who had a shopping experience either from the brick-and-mortar or web fronts. Therefore, participants were required to participate in any omnichannel shopping for fashion accessories. A quantitative survey research strategy was used to gather the primary data for this study. Data was gathered from the study's research population in Thailand using a stratified random sampling strategy. The data was collected from major towns in Thailand (i.e. Bangkok, Chonburi, Phuket, KhonKaen, and Chiang Mai) which were selected using convenience sampling, following the fact that people living in these towns were customers in fashion retail sector. Data collection for the study occurred from 16 October 2023, to February 29, 2024. The researcher utilized a structured questionnaire administered to participants, who provided responses using a Likert scale at five-point. The questionnaire was distributed online via the Google Forms during the data collection process. To meet the inclusion criterion, respondents were required to have used an omnichannel platform at least once in their shopping activities. The latent variables in the study were derived from the conceptual framework and literature review, ensuring their alignment with the research objectives. The measurement items, which captured the constructs under investigation, were developed based on previous studies and subsequently modified to suit the specific context of the fashion retail sector in Thailand. Careful adjustments were made to certify the appropriateness and relevance of the items measured within the research context based on the recommendation of three experts who validated the research instrument. The questions regarding perceived value, perceived enjoyment and perceived ease of use were adapted from Jia et al. [67], de Souza et al. [72], and Kim et al. [73]. Wolfinbarger and Gilly [95] and Neslin et al. [96] provided the omnichannel customer experience and integrated customer service scales. The measurement items for customer satisfaction were adapted from Wolfinbarger and Gilly [95], Hausman and Siekpe [97], and Lin and Wang [98]. Integrated promotions scales were developed from Porcu et al. [99] and Horstmann [100]. Integrated order filfilment questions were adapted from Wilfinbarger and Gilly [95], Nanda and Patnaik [92], and Lee et al. [101], while Integrated transactions questioned were revised from Dastane et al. [102], Bauer et al. [103], and Angelidou et al. [104]. The targeted sample size was 700 respondents. As a result, 700 questionnaire copies were circulated, from which 537 participants correctly completed the questionnaire. After reviewing and cleaning the returned questionnaires for missing data and outliers, the appropriate responses were 509, making this the sample size.

### 3.1 Measures

The methodology utilizes a detailed empirical analysis by using structural equation modeling, starting within the context of UTAUT constructs, linking customer satisfaction and omnichannel shopping, and the need to validate the model in the retail fashion industry in Thailand. The data collected for this study underwent rigorous analysis utilizing the AMOS software. Initially, the validity and reliability construct were assessed to confirm the data accuracy and quality. Discriminant validity and convergent validity were evaluated through a Confirmatory Factor Analysis (CFA). This data analysis for the study provided interesting perceptiveness into the reliability and distinctiveness of the measurement items. Furthermore, the fitness of the model was evaluated utilizing multiple fitness tests including Root Mean Square Error of Approximation (RMSEA), Comparative Fit Index (CFI), Tucker-Lewis Index (TLI), and Normed Fit Index (NFI). The tests conducted evaluated how well the proposed conceptual framework fits the collected data, indicating the model's adequacy. To investigate the correlations linking the study variables as the conceptual framework outlined, structural equation modeling was deployed. This analysis assessed the strength and significance of the paths between variables. These correlations were analyzed using the beta coefficients and the significance levels to facilitate evaluating the hypotheses and contextualize the effects of the independent variables on the dependent ones.

## 4 Results

### 4.1 Demographic characteristics

The demographic characteristics of the respondents take awareness of factors including the gender, age, and educational qualifications they possess. An analysis of the gender data from the participant demographic profile illustrates that majority of respondents were female, comprising 53.8 %, as evidenced by the analyzed empirical research data. In contrast, the males were about 39.5 % of the study respondents as observed. The interesting data from the gender dynamics was the representations of those who neither identified as male or female accounting for 6.7 % of the population. The capture of this vital demographic will help retailers to identify their preferences when making marketing decisions on fashion items to be promoted. As per the age distribution, the largest observed group were those in the age range of 31–40 (37.3 %). They were closely followed by the 41–50 (31.8 %) age range. Conversely, the 51–60 (7.7 %) age range represented the smallest proportion of the research respondents. With respect to the educational credentials, the analysis revealed the respondents predominantly possessed a bachelor's degree (44.6 %) of the surveyed study sample. Additionally, individuals with a certificate or diploma accounted for 38.3 % of the participants, reflecting the second-largest educational category. The years of experience using omnichannels revealed that more of the respondents started using omnichannels less than one year ago (34.6 %), while those with over five years of experience were the least comprising 9.2 % of the respondents. Table 1 summarizes the demographic data.

**Table 1:** Demographic characteristics.

Variable	Categories	Frequency	Percent
Gender	Male	201	39.5
	Female	274	53.8
	Other	34	6.7
Age	18–30 Years	118	23.2
	31–40 Years	190	37.3
	41–50 Years	162	31.8
	51–60 Years	39	7.7
Education	High School/or Lower	38	7.5
	Certificate-Diploma	195	38.3
	Bachelor's	227	44.6
	Post-Graduate/or Higher	49	9.6
Experience (years)	Less than 1 Year	176	34.6
	2–3 Years	134	26.3
	3–4 Years	152	29.9
	5 Years & above	47	9.2

### 4.2 Model evaluation

The fitness of the study model was appraised utilizing CFA to test how the model fitted the data. The research reliability was evaluated employing convergent reliability and Cronbach's alpha. Similarly, validity was assessed utilizing discriminant validity, AVE, and standardized factor loadings. The required threshold for the standardized factor loadings and average variance extracted was 0.5. While assessing the factor loadings, IOF3 was below the required threshold of 5.0, and therefore was removed. The adjusted model was re-run, and the results were satisfactory. The adjusted model values of the factor loadings varied from 0.521 to 0.830, while the average variance extracted values ranged from 0.503 to 0.630, which fulfilled the threshold requirements set. For reliability test, Cronbach's alpha values varied from 0.759 to 0.878, while convergent reliability oscillate between 0.769 and 0.880. The values indicated that there was high internal consistency reliability. These values were within the required level of >0.7 [105], as Table 2 shows, while Table 3 presents the descriptive statistics of the Likert variables.

**Table 2:** Model evaluation.

Latent Variables	Items	Factor loadings	CR	AVE	Cronbch's alpha
Perceived Enjoyment	EN1	0.751	0.876	0.585	0.878
	EN2	0.722			
	EN3	0.801			
	EN4	0.762			
	EN5	0.786			
	ICS1	0.715	0.854	0.54	0.855
Integrated Customer Service	ICS2	0.752			
	ICS3	0.724			
	ICS4	0.758			
	ICS5	0.723			
Integrated Order Fulfillment	IOF1	0.706	0.747	0.63	0.767
	IOF2	0.755			
	IOF4	0.521			
	IOF5	0.615			
Integrated Promotions	IP1	0.73	0.872	0.577	0.874
	IP2	0.811			
	IP3	0.764			
	IP4	0.751			
	IP5	0.739			
Integrated Transactions	IT1	0.686	0.837	0.508	0.839
	IT2	0.688			
	IT3	0.736			
	IT4	0.737			
	IT5	0.714			
Omni-channel Customer Experience	OCE1	0.763	0.868	0.568	0.869
	OCE2	0.781			
	OCE3	0.771			
	OCE4	0.725			
	OCE5	0.725			
Omni-channel Satisfaction	OS1	0.738	0.879	0.592	0.88
	OS2	0.764			
	OS3	0.802			
	OS4	0.758			
	OS5	0.785			
Perceived Ease of Use	PEU1	0.562	0.833	0.503	0.855
	PEU2	0.704			
	PEU3	0.83			
	PEU4	0.644			
	PEU5	0.776			
Perceived Value	PV1	0.787	0.884	0.604	0.885
	PV2	0.763			
	PV3	0.806			
	PV4	0.74			
	PV5	0.788

**Table 3:** Descriptive statistics of the Likert scales variables.

Variable	Mean	Std. Deviation	Minimum	Maximum
Perceived Enjoyment (EN)	3.80	0.85	1	5
Integrated Customer Service (ICS)	3.80	0.86	1	5
Integrated Order Fulfillment (IOF)	3.75	0.86	1	5
Integrated Promotions (IP)	3.90	0.80	1	5
Integrated Transactions (IT)	3.84	0.83	1	5
Omnichannel Customer Experience (OCE)	3.75	0.84	1	5
Omnichannel Satisfaction (OS)	3.76	0.84	1	5
Perceived Ease of Use (PEU)	3.65	0.94	1	5
Perceived Value (PV)	3.79	0.86	1	5

In addition, the model fitness was evaluated using various tests. The tests conducted included the chi-square values, the root mean square error of approximation (RMSEA), the comparative fit index (CFI), and the Tucker–Lewis index (TLI). The results indicated that the CMIN/DF = 2.875, which satisfied the required threshold of <3.0. The CFI = 0.913, TLI = 0.904, IFI = 0.914, NFI = 0.866; and GFI = 0.834. The fitness indices for CFI, TLI and IFI were above 0.9, the required threshold, while the indices for NFI and GFI were above the satisfactory threshold of 0.8 [106,107]. These findings confirmed that the required threshold for model fitness was satisfied.

These descriptive statistics provide an overview of the central tendency and dispersion of the responses on the Likert scale, giving a better understanding of the respondents' attitudes towards each variable. It indicates that respondents generally had positive attitudes towards various aspects of the omnichannel shopping experience. The mean scores for all variables were above 3.5 on a 5-point scale, with the highest being Integrated Promotions (IP) averaging 3.90. The least was Perceived Ease of Use (PEU) with an average of 3.65. This indicated that generally, the respondents were generally satisfied with their various experiences considered in the study. The standard deviations ranged from 0.83 to 0.94, reflecting moderate variability in responses. Minimum and maximum scores ranged from 1 to 5, indicating the full range of possible responses was utilized. These statistics highlight a generally favorable perception of omnichannel elements among respondents.

### 4.3 Hypotheses evaluation

**Fig. 2:**
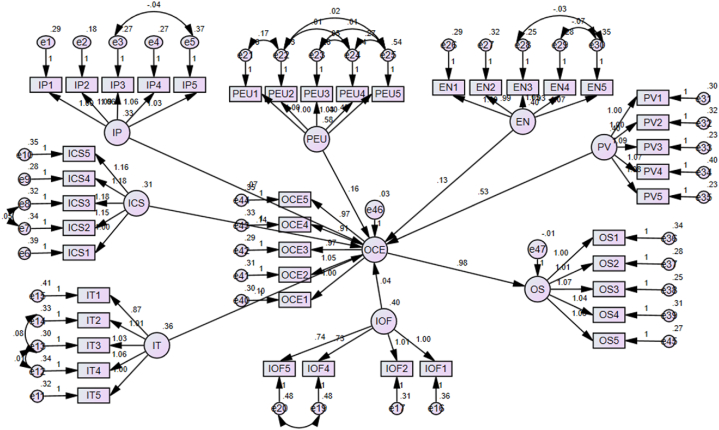
Hypothesis evaluation.

The study hypotheses were evaluated by running a Structural Equation Modelling (SEM) model. The results are presented in Table 4 and Fig. 2. The results indicated that the path coefficient between perceived ease of use and Omni-channel customer experience was positive and significant (β = 0.163, p = 0.000), supporting hypothesis 1. The path coefficient between enjoyment and Omni-channel customer experience was positive and significant (β = 0.133, p = 0.000), supporting hypothesis 2. The path coefficient between perceived value and Omni-channel customer experience was positive and significant (β = 0.533, p = 0.000), supporting hypothesis 3. The path coefficient between integrated promotions and Omni-channel customer experience was positive and significant (β = 0.070, p = 0.003), supporting hypothesis 4. The path coefficient between integrated customer service and Omni-channel customer experience was positive and significant (β = 0.140, p = 0.000), supporting hypothesis 5. The path coefficient between integrated transactions and Omni-channel customer experience was positive and significant (β = 0.103, p = 0.000), supporting hypothesis 6. The path coefficient between integrated order fulfillment and Omni-channel customer experience was positive and insignificant (β = 0.036, p = 0.115), rejecting hypothesis 7. The path coefficient between Omni-channel customer experience and omnichannel satisfaction was positive and significant (β = 0.976, p = 0.000), supporting hypothesis 8.

**Table 4:** Hypothesis evaluation.

Hypothesis Relationship				Estimate	S.E.	t-value	P-value
H1	PEU	→	OCE	0.163	0.023	7.098	***
H2	EN	→	OCE	0.133	0.023	5.756	***
H3	PV	→	OCE	0.533	0.043	12.491	***
H4	IP	→	OCE	0.07	0.024	2.964	**
H5	ICS	→	OCE	0.14	0.027	5.209	***
H6	IT	→	OCE	0.103	0.024	4.228	***
H7	IOF	→	OCE	0.036	0.023	1.577	0.115
H8	OCE	→	OS	0.976	0.088	11.091	***

Note: *** = significant at 99 % confidence level, ** = significant at 95 % confidence level; PV = perceived value, EN = perceived enjoyment, PEU = perceived ease of use, OS = omni-channel satisfaction, OCE = omni-channel customer experience, IT = integrated transactions, IP = integrated promotions, EN = perceived enjoyment, IOF = Integrated Order Fulfillment, ICS = integrated customer service, OS = omni-channel satisfaction.

In addition to direct effect, the indirect effect (mediating effect of omnichannel customer experience) was evaluated. Bootstrapping technique was adopted for mediating analysis. The study analyzed the mediating effect omnichannel customer experience on the effect of all the independent variables (perceived value, perceived enjoyment, perceived ease of use, integrated promotion, integrated customer service, integrated transaction, and integrated order fulfillment) on omnichannel customer satisfaction. The results are summarized in Table 5. The results indicated that omnichannel customer experience significantly mediates the effect of perceived ease of use on omnichannel customer satisfaction (β = 0.160, p < 0.05). Omnichannel customer experience significantly mediates the effect of perceived enjoyment on omnichannel customer satisfaction (β = 0.130, p < 0.05). Omnichannel customer experience significantly mediates the effect of perceived value on omnichannel customer satisfaction (β = 0.130, p < 0.05). Omnichannel customer experience significantly mediates the effect of integrated customer service on omnichannel customer satisfaction (β = 0.137, p < 0.05). Omnichannel customer experience significantly mediates the effect of integrated order fulfillment on omnichannel customer satisfaction (β = 0.137, p < 0.05).

**Table 5:** Mediating effects of omnichannel customer experience.

Hyp. No.	Path					Total Effect	Direct Effect	Indirect Effect	Hypothesis confirmed?
H9a	PEU	→	OCE	→	OS	0.160	0.163	0.160**	Supported
H9b	EN	→	OCE	→	OS	0.130	0.133	0.130**	Supported
H9c	PV	→	OCE	→	OS	0.520	0.533	0.520**	Supported
H9d	IP	→	OCE	→	OS	0.060	0.070	0.069	Not Supported
H9e	ICS	→	OCE	→	OS	0.137	0.140	0.137**	Supported
H9f	IT	→	OCE	→	OS	0.100	0.103	0.100**	Supported
H9g	IOF	→	OCE	→	OS	0.035	0.036	0.035	Not Supported

Note: *** = significant at 99 % confidence level, ** = significant at 95 % confidence level; PV = perceived value, EN = perceived enjoyment, OS = omni-channel satisfaction, OCE = omni-channel customer experience, IT = integrated transactions, IP = integrated promotions, PEU = perceived ease of use, IOF = integrated order fulfillment, ICS = integrated customer service, OS = omni-channel satisfaction.

## 5 Discussions

The purpose of this study was to examine consumer perceptions regarding the omnichannel shopping experience and satisfaction in the fashion retail sector in Thailand. The study was considered vital and interesting, bearing in mind that omnichannel retailing is quite a new concept that has revolutionized the retail sector. It creates a mechanism through which customers can access the purchase experience through various platforms such as traditional storefronts, online marketplace, and social media channels. This research used two perspectives to investigate how omnichannel influence shoppers' experiences and satisfaction – aspects borrowed from the UTAUT model and the omnichannel predictors. The study's findings are insightful regarding the demographic attributes of the participants and offer perspectives that illuminate and assess the study framework and hypotheses as proposed.

The demographic characteristics of the respondents revealed interesting insights that delineate the respondent gender distinctions; most of the respondents were female, comprising 53.8 %, while males accounted for 39.5 %. Those who identified as other which include other non-gender conforming individuals accounted for 6.7 % of the respondents. This gender distribution suggests that females are important when discussing the fashion retail sector in Thailand; this highlights the importance of understanding their perspectives on omnichannel shopping satisfaction, and also reveals the importance of non-gender conforming consumers. While their number may not be comparable to those identifying as male and females, future studies will keep revealing interesting and vital information about their preferences and choices when it comes to fashion and other retail items. Regarding age, the largest proportion of the study respondents fell within the 31–40 years bracket. They were closely followed by the 41–50 years age group. This indicates that individuals considered middle-aged actively engage in omnichannel shopping in the fashion retail sector in Thailand. The least represented age group was those in the 51–60 age bracket. This suggests that older adults/seniors may be less agreeable to adopting omnichannel shopping practices.

These findings reiterate the rationale for fashion retailers to tailor their omnichannel strategies and messages to cater to different age groups and demographic segments. The educational qualifications of the study demographic throw more interesting insights that would be valuable to retailers, marketers and other stakeholders in product development chain interested in adopting omnichannel marketing in their business activities. It is noted that 45.9 percent of respondents completed a bachelor's, followed by those who had a certificate or diploma (35.1 %), making those with formal education certification the overwhelming majority, comprising 81 % of respondent demographic. The link between educational qualification and the use of omnichannel shopping suggests a correlation between education level and the likelihood of utilizing omnichannel shopping in the fashion retail sector in Thailand and, by extension, omnichannel shopping in general. Fashion retailers should consider this when designing and implementing omnichannel strategies, ensuring that they cater to the preferences and needs that will draw in the market segment of educated consumers.

Moving on to the evaluation of the research model, CFA assessed the model's fitness, and the results demonstrated satisfactory model fit, as evidenced by the acceptable standardized factor loadings and AVE values. These findings indicate that the research model accurately reflects the underlying relationships among the constructs. In estimating Cronbach's alpha and convergent reliability, reliability evaluation revealed high internal consistency among the constructs. The obtained Cronbach's alpha values obtained varied from 0.759 to 0.878, surpassing recommended limit of 0.7.

Additionally, convergent reliability values fluctuate from 0.769 to 0.880, further supporting the measurement model's reliability. These results affirm the study instrument's robustness and data reliability. The discriminant validity was evaluated utilizing Fornell and Larcker metric [108]. It demonstrated that the research model's constructs were distinct. The square root of each construct's AVE exceeded required correlation to other constructs, indicating that constructs measure unique aspects of the phenomena under investigation. This confirms the discriminant validity among the latent variables and strengthens the research model's validity. The hypotheses evaluation utilizing Structural Equation Modeling (SEM) yielded significant results for several relationships. Perceived ease of use, enjoyment, perceived value, integrated promotions, integrated customer service, and integrated transactions significantly and influenced omnichannel customer experience positively. They aligned with previous studies highlighting how important these factors are in shaping customers' experiences and enjoyment in omnichannel usage.

The findings disclosed that perceived value strongly influenced the omnichannel customer experience, followed by perceived ease of use and perceived enjoyment. The aspect of perceived value includes access to relevant shopping information, ease of purchase, assistance in decision-making, and smart shopping. These findings are supported by Mahadevan and Joshi [49] and Ramadan and Nsouli [77], who indicated that perceived value is critical in determining customers' attitudes toward omnichannel shopping. Additionally, Sousa et al. [51] found that perceived value was an essential aspect of sustainability in Brazilian omnichannel retail. The ease of utility is a fundamental aspect which influences the customers' experiences with omnichannel, including aspects such as how easy it is to make purchases and maneuver among the integrated channels. These results are supported by Cattapan and Pongsakornrungsilp [53], whose research indicated that the perceived ease of use of omnichannel shopping was positively linked to millennial purchase intention for fashion retailers. Perceived enjoyment was discovered to be an important element in adoption of omnichannel shopping in the fashion retailing in Thailand because it influenced people's attitudes toward omnichannel shopping. Similar to the findings of this study, Cotarelo et al. [11] found that enjoyment significantly predicted customer satisfaction and loyalty in omnichannel shopping.

For the omnichannel factors, this research found that integrated promotions, integrated customer services, and integrated transactions significantly and positively affected omnichannel customer experience. However, it is worth noting that the correlation of integrated order fulfillment with omnichannel customer experience was found to be insignificant. While it is clear that other elements critically affect the omnichannel experience for Thai shoppers in Thai fashion retail sector, it is evident that the integrated order fulfillment component deserves more attention if the intention is to maximize the positive effect it has on customer satisfaction. Fashion retailers in Thailand can improve customers' satisfaction with the omnichannel shopping process by implementing integrated promotional strategies that deliver a consistent message, which is timely to deliver a seamless customer experience. For emphasis, to achieve consistency in promotion outcomes, Thai fashion retailers should effectively utilize multiple communication channels and provide customers with engaging content with specifics at every contact point. Similarly, fixing communication and system flaws on the omnichannel platform, expanding problem-solving skills, and guaranteeing a pleasant purchasing experience are all facilitated by an integrated customer service approach. This, in turn, can contribute to increased customer satisfaction, repeated purchases, and positive word-of-mouth recommendations for the fashion retailer sector in Thailand which can enhance sustainability of the business.

The study found that integrated transactions contribute to satisfaction of customers in omnichannel shopping. This observation is showcased in features that highlight purchase history, access to purchase details, receiving recommendations for future purchases and enjoying personalized services for both online and offline customers. When customers have a consistent payment process it builds trust and confidence in fashion retailers ultimately influencing how they perceive them and their willingness to engage in omnichannel shopping. Consequently, an integrated transactions approach plays a role in shaping customers satisfaction and overall experience when using omnichannel platforms within the fashion industry.

The finding that integrated order fulfillment (IOF) has an insignificant impact on omnichannel customer experience may appear contradictory to the goals of implementing an omnichannel strategy. To address this, it is important to explore and compare relevant literature to provide context and possible explanations. Several studies have highlighted the importance of seamless order fulfillment in enhancing customer satisfaction in omnichannel retailing. For instance, Gao et al. [26] emphasize that effective order fulfillment is crucial for a positive omnichannel experience, as it directly affects customer perceptions of reliability and convenience. Similarly, Mofokeng and Tan [109] discuss how efficient order fulfillment processes can significantly influence customer satisfaction and loyalty. However, some research suggests that the impact of order fulfillment may vary depending on the context and other factors. Davis-Sramek et al. [110], supported by Nanda and Patnaik [92], found that while order fulfillment is essential, other aspects, such as customer service and personalized interactions, can substantially impact overall customer experience; this aligns with the current study's findings, where integrated customer service and personalized transactions significantly positively affected the omnichannel experience. The current study's results suggest that, in Thai fashion retail, other factors such as perceived value, ease of use, and integrated customer service play more prominent roles in shaping the omnichannel experience. This could be due to specific cultural or market characteristics where customers prioritize these aspects over order fulfillment efficiency.

The SEM results indicate that perceived ease of use (PEU), perceived enjoyment (EN), perceived value (PV), integrated promotions (IP), integrated customer service (ICS), and integrated transactions (IT) all have significant positive impacts on omnichannel customer experience (OCE), which in turn significantly affects omnichannel satisfaction (OS), and these are supported by the literature [1,23,111,112]. This path analysis demonstrates the interconnected nature of these factors and underlines the complex relationship existing between the different variables in the omnichannel experience; while integrated order fulfillment did not show a significant direct effect, its role should not be dismissed entirely. It may still contribute indirectly to customer satisfaction through other mediating factors, such as perceived value or customer service, as pointed out by Nanda and Patnaik [92]. In hindsight, the study's findings are consistent with some literature suggesting the omnichannel customer experience's multifaceted nature, where factors beyond order fulfillment play critical roles [1,75].

Customer services that are integrated involve aspects such as accepting complaints, returns and repairs, providing post purchase services across all channels and offering repair and claim services regardless of the channel used. Fashion retailers can enhance customer satisfaction and loyalty by providing a uniform and smooth user support experience on multi-channels. These integrated customer service practices contribute to the intensity and shopping value that are crucial determinants of consumer happiness and loyalty [84]. Furthermore, the study affirms a significant and positive correlation relating omnichannel customer experience and satisfaction with using the channels. This underlines the importance of delivering a seamless and integrated shopping involvement on multiple platforms to enhance customer satisfaction. Chen et al. [87] supports these findings by highlighting that the quality of channel integration significantly influences customers' responses regarding omnichannel ecommerce. Fashion merchants are in a position to enhance customers’ satisfaction by offering a seamless, personalized, and interactive platform experience to customers. This can be achieved through consistent branding, tailored recommendations, efficient customer support, and cohesive service delivery. Prioritizing the omnichannel customer experience enables fashion retailers in Thailand to differentiate themselves, establish strong customer relationships, and ultimately drive customer satisfaction and loyalty. Further, the omnichannel customer experience was found to serve as a pivotal mediator, linking various factors such as perceived ease of use, enjoyment, value, integrated customer service, and integrated order fulfillment to overall customer satisfaction. It implied that providing customers with seamless and convenient shopping experiences across multiple channels positively influences their overall satisfaction. Additionally, these findings stress the importance of empowering frontline staff with the requisite skills and resources to deliver consistent and high-quality service throughout the customer journey.

### 5.1 Recommendations and implications

This research has both theoretical and empirical implications. Theoretical implications stem from developing a framework that integrates the UTAUT model with omni-channel-related aspects and examines their effects on the omnichannel customer experience and satisfaction. Analyses have highlighted how this contribution is significant in augmenting the reviewed studies. From the UTAUT model perspective, perceived enjoyment, perceived value and perceived ease of use emerged as significant factors. On the other hand, from the omnichannel perspective, integrated promotions, integrated customer service, and integrated transactions were identified as significant aspects. The empirical implications of this study revolve around the critical exploration of customers' perspectives regarding omnichannel shopping, specifically in terms of their satisfaction and experience within the fashion retail sector. The study established the several complex aspects influencing Thailand's fashion retail industry; customers' shopping experience and satisfaction were found to be contingent upon various factors. Importantly, perceived value, perceived ease of use, integrated transactions, integrated promotions, enjoyment, and integrated customer service were identified as vital factors in enhancing omnichannel customer satisfaction. The findings of this study add to our theoretical understanding by establishing an integrated framework, highlighting the significant factors from both the UTAUT model and omnichannel perspectives. Furthermore, it offers empirical insights into customers' perspectives, satisfaction, and experiences in the fashion retail sector in Thailand. The identified factors play a crucial role in shaping customers' satisfaction within an omnichannel context.

Several suggestions for improving satisfaction can be deduced based on the results of this study regarding applying omni-channels in the fashion sector in Thailand and other similar applicable industries/regions/countries. Firstly, market players should prioritize enhancing the overall customer experience with omnichannels. Critical aspects of the customer experience that influence their satisfaction with omni-channels include the level of connectivity, flexibility, integration of all shopping channels, and increased connectivity within the omnichannel system. By focusing on these aspects, fashion retailers can create a seamless and satisfying omnichannel experience for customers. Furthermore, specific factors should be emphasized to improve the customer experience within omni-channels. The research suggests that the three key variables of perceived ease of use, value and enjoyment should be enhanced. If customers foresee advantages to adopting omnichannel in purchase decisions, and they find the system user-friendly, it will greatly enhance their customer experience and increase the adoption rate as observed from the fashion retail experience in Thailand. Therefore, it is crucial to focus on these specific perspectives and ensure they meet customer expectations. Additionally, to enhance omnichannel operations further, retailers should work on improving integrated transactions, promotions, and customer service.

Fashion retailers, to achieve customer satisfaction, should focus on integrating payment options and channels ensuring consistent and seamless promotional activities, across all channels and delivering a cohesive and effortless customer support experience at every point of contact. The key for Thai retailers, will be to strengthen these areas to deliver a remarkably enhanced and effective omnichannel operations, leading to increased customer satisfaction which leads to repeat purchase. Part of the researcher's recommendation toward refining omnichannel shopping experiences for fashion customers in Thailand are to prioritize attributes such as enriched connectivity of the platform, flexibility to afford users with more purchasing options, integration of different interrelated platforms such as the ability to incorporate social media (Facebook, Twitter, Instagram, etc.) plugins and card payment capabilities and pay on delivery (POD) options within the omnichannel framework. Fashion retailers in Thailand should prioritize improving customer engagement and extending their domestic and international reach by enhancing the value perception of their omnichannel platforms', customer satisfaction and ease of use. Optimizing omnichannel operations requires perfecting the system of synchronization of all sales, marketing campaigns, promotion campaigns and customer service activities across all channels with verified business prospects.

### 5.2 Limitations and directions for further studies

Like any empirical examination, there are limitations to acknowledge. Firstly, due to the aim of collecting quantitative data from a diverse sample population, the small sample size could have resulted in limited statistical power [113], potentially leading to insignificant or erroneous findings. However, despite the small sample size, the significant findings obtained in this study provide valuable insights into customer satisfaction in the context of omnichannels within Thailand's fashion retail industry. Secondly, although efforts were made to control variations by focusing on one industry in Thailand, it is important to recognize that the applicability of the results may be restricted to the fashion retail in Thailand. Further research is warranted to incorporate a wider range of industry sectors, encompassing both developing and developed economies, as well as considering the influence of sociopolitical influences on organizational experiences [114].

Lastly, addressing the potential presence of common method bias in customer satisfaction responses is essential. Despite encountering challenges in obtaining direct responses from fashion retail customers and considering that the other variables were derived from a combination of buyer-seller responses, we deemed the customer satisfaction responses appropriate and valid for utilization in this study. While these limitations should be acknowledged, they do not diminish the quality and significance of the insights generated by analyzing customer satisfaction within the omnichannel context of Thailand's fashion retail business. Future endeavors could target overcoming these limitations, while expanding the investigation scope investigation for a more comprehensive understanding of customer satisfaction in diverse settings. To fully comprehend the omnichannel shopping experience, future research should delve into customers' perspectives and their satisfaction levels. Investigating how customers perceive and engage with various omnichannel touchpoints including virtual platforms, apps, and in-person front stores, can proffer valuable insights into the factors that drive customer satisfaction.

Drawing from the UTAUT model and specific omnichannel aspects, future studies should examine the factors significantly influencing omnichannel shopping satisfaction. Some of the dynamics that influence client satisfaction include perceived enjoyment, integrated promotions, perceived ease of use, and integrated customer service. Studying the relationships between these factors can offer more perceptiveness on their relevance and cumulative effect on consumers' satisfaction. A vital discernment emanating from the study is that executing integrated order fulfillment in Thailand's fashion retail sector has minimal effect on the prevalent omnichannel experience for consumers when shopping. Future studies should conduct more elaborate and in-depth examinations to understand the underlying rationales that account for integrated order fulfillment having a limited impact among Thailand fashion consumers utilizing omnichannel platforms. Understanding the potential areas, where omnichannel marketing in Thai fashion retail can be enhanced will assist in exploring the concomitant intricacies of executing integrated order fulfillment systems in the sector. Identifying these lacunas, by fashion retailers will enable storefronts to improve their order fulfillment procedures and, in turn, their customers' experiences.

The author infers that perceived value, enjoyment and ease of use should be emphasized. Thai retailers can focus on communicating the core value propositions of their omnichannel offerings while similarly guaranteeing an intuitive and enjoyable user interface. To better serve their customers, Thai storefronts should work toward integrating all aspects of their business, including sales, marketing, and support, to cohesively work as a single entity interacting with each other. Thai businesses can provide a unified and satisfying consumer shopping experience through an effortless integration of these elements across the omnichannel channel platform of activities. For emphasis the study recommends that retailers and shoppers alike aspire to enhance the omnichannel purchasing process while noting that this suggestion is not restricted to the Thai fashion retail sector. It is relevant for any sector that employs omnichannel marketing, wherever it may be practiced.

## 6 Conclusions

This research significantly contributes to the existing body of knowledge on omnichannel retailing by providing an exhaustive analysis of customer satisfaction in the Thai fashion retail sector. Utilizing the Unified Theory of Acceptance and Use of Technology (UTAUT) model, key determinants such as perceived ease of use, perceived enjoyment, perceived value, integrated promotions, integrated customer service, and integrated transactions that positively influence the omnichannel customer experience are identified; importantly, it highlights the critical role of integrated order fulfillment, which, contrary to conventional wisdom, was found to have an insignificant impact on customer satisfaction, challenging prevailing assumptions and underscoring the need for further theoretical exploration. By the inclination of focusing on the Thai market, the study fills a geographical gap in the literature, offering a better understanding into the unique consumer behaviors and preferences are influenced by the application of omnichannel in a rapidly growing Southeast Asian flagship with support from prior studies [54,58]. The methodological rigor, employing SEM, provides valid and reliable findings that can inform both academic discourse and practical applications. Fashion retailers can utilize these insights to refine and develop their omnichannel strategies, enhancing customer satisfaction and loyalty. Invariably, this study advances theoretical understanding and provides actionable recommendations for practitioners aiming to optimize their omnichannel retail operations.

As evidenced by the study findings in Thailand's thriving fashion industry, the omnichannel marketing strategy is becoming increasingly integral to the sustenance of retail operations as the business landscape evolves with more roles for technology. The whole prospect of this omnichannel concept is still being explored with the availability of new data on consumer behavior, and the introduction of innovation in society ushers in fronts to conquer for retail fashionpreneurs. Customer satisfaction on omnichannel platforms is the centerpiece that substantially affects the adoption and deployment of these systems. With the witnessed accentuation of omnichannel utilization in the fashion retail sector, we can attribute this to the expanding digital integration in Thai land and the rising cravings of customers for comfort and customization, which we propose as 2Cs. The investigation was hinged on the postulations of the UTAUT model; it was investigated to determine its congruence and applicability in assessing different omnichannel aspects with data emanating from the fashion retail sector of Thailand. From the empirical study the level of satisfaction with the technology significantly impacts the omnichannel experiences of Thai fashion retail customers. Customers' satisfaction with their omnichannel shopping experience was further segmented to reveal that factors including perceived ease of use, perceived enjoyment, integrated promotions, integrated customer service, and integrated transactions were crucial. Nonetheless, the empirical analysis also revealed that integrated order fulfillment had no significant influence on shoppers' omnichannel experience. While this is new information, it harps on the need for further investigation into why customers feel that order fulfillment does not hamper their omnichannel experience.

Finally, because the research concentrated on the Thai fashion retail industry, the findings have limited generalizability. Though significant data unique to this situation is provided by the results, they might not immediately apply to other sectors or geographic areas with distinct consumer behaviors and market dynamics. Unique cultural and economic elements of Thailand may affect how customers view and prioritize omnichannel buying. Still, the fundamental ideas about the value, perceived ease of use, and integrated services may provide pertinent information for different markets, indicating that more cross-cultural study is required to confirm and broaden these conclusions.

## Funding

This work was financially supported by KMITL Business School, 10.13039/501100007120King Mongkut's Institute of Technology Ladkrabang, Bangkok 10520, Thailand.

## Ethics statement

To ensure ethical compliance, the study was reviewed and received an exemption from the Ethics Committee of King Mongkut's Institute of Technology Ladkrabang; with approval number EC-KMITL_66_104. The respondents and the experts involved in the validation of the research instrument all provided informed consent to participate in the research.

## Disclosure statement

The author declares that they have no known competing personal relationships or financial interests that could have appeared to influence the findings reported in this research.

## Data availability statement

The data used in this research are available upon request from the author.

## CRediT authorship contribution statement

**Bilal Khalid:** Writing – review & editing, Writing – original draft, Visualization, Validation, Supervision, Software, Resources, Project administration, Methodology, Investigation, Funding acquisition, Formal analysis, Data curation, Conceptualization.

## Declaration of competing interest

The authors declare that they have no known competing financial interests or personal relationships that could have appeared to influence the work reported in this paper.

## References

[1] Riaz H., Baig U., Ahmed H. (2022). Factors effecting omnichannel customer experience: evidence from fashion retail. Information.

[2] Shi S., Wang Y., Chen X., Zhang Q. (2020). Conceptualization of omnichannel customer experience and its impact on shopping intention: a mixed-method approach. Int. J. Inf. Manag..

[3] Chevalier S. (2022). https://www.statista.com/statistics/379046/worldwide-retail-e-commerce-sales/.

[4] Bălan C., Chaffey D. (2014). Digital business and e‐commerce management: strategy, implementation and practice. Manag. Market..

[5] Sorkun M.F., Yumurtacı Hüseyinoğlu I.O., Börühan G. (2020). Omni-channel capability and customer satisfaction: mediating roles of flexibility and operational logistics service quality. Int. J. Retail Distrib. Manag..

[6] Merritt K., Zhao S. (2020). An investigation of what factors determine the way in which customer satisfaction is increased through omni-channel marketing in retail. Adm. Sci..

[7] Kiba-Janiak M. (2014). The use of mobile phones by customers in retail stores: a case of Poland. Economics & Sociology.

[8] Sloboda L., Dunas N., Limański A. (2018). Contemporary challenges and risks of retail banking development in Ukraine. Banks Bank Syst..

[9] Chatzoglou P., Chatzoudes D., Savvidou A., Fotiadis T., Delias P. (2022). Factors affecting repurchase intentions in retail shopping: an empirical study. Heliyon.

[10] Kaur K., Bakar E.A., Singh J. (2020). 20th Kuala Lumpur International Business, Economics and Law Conference.

[11] Cotarelo M., Fayos T., Calderón H., Mollá A. (2021). Omni-channel intensity and shopping value as key drivers of customer satisfaction and loyalty. Sustainability.

[12] Jiang Y., Stylos N. (2021). Triggers of consumers' enhanced digital engagement and the role of digital technologies in transforming the retail ecosystem during COVID-19 pandemic. Technol. Forecast. Soc. Change.

[13] Kráľ Š., Fedorko R., Štofejová L., Kizák M. (2022). An analytical view of consumers' purchasing behaviour in terms of e-commerce during COVID-19. Polish Journal of Management Studies.

[14] Streimikiene D. (2022). Energy poverty and impact of Covid-19 pandemics in Visegrad (V4) countries. Journal of International Studies.

[15] Hole Y., Pawar M.S., Khedkar E.B. (2019). Omni channel retailing: an opportunity and challenges in the Indian market. J. Phys. Conf..

[16] Thaichon P., Phau I., Weaven S. (2022). Moving from multi-channel to Omni-channel retailing: special issue introduction. J. Retailing Consum. Serv..

[17] (2022). Statista, Thailand: Apparel Market Revenue 2013-2026.

[18] Cortiñas M., Chocarro R., Elorz M. (2019). Omni-channel users and omni-channel customers: a segmentation analysis using distribution services. Spanish Journal of Marketing-ESIC.

[19] Basuki B., Szczepańska-Woszczyna K., Rajiani I., Widyanti R., Kot S. (2022). Working from home arrangement in delivering public service during the COVID-19 pandemic: innovation or irritation?. Administratie si Management Public.

[20] Hendalianpour A., Fakhrabadi M., Sangari M.S., Razmi J. (2022). A combined benders decomposition and Lagrangian relaxation algorithm for optimizing a multi-product, multi-level omni-channel distribution system. Sci. Iran..

[21] Venkatesh V., Thong Y.Y.L., Xu X. (2012). Consumer acceptance and use of information technology: extending the unified theory of acceptance and use of technology. MIS Q..

[22] Hoyer W.D., Kroschke M., Schmitt B., Kraume K., Shankar V. (2022). Transforming the customer experience through new technologies. J. Interact. Market..

[23] Sharma N., Fatima J.K. (2024). Influence of perceived value on omnichannel usage: mediating and moderating roles of the omnichannel shopping habit. J. Retailing Consum. Serv..

[24] Jo H., Bang Y. (2024). Navigating the omnichannel landscape: unraveling the antecedents of customer loyalty. Sage Open.

[25] Aslam H., Waseem M., Muneeb D., Ali Z., Roubaud D., Grebinevych O. (2023). Customer integration in the supply chain: the role of market orientation and supply chain strategy in the age of digital revolution. Ann. Oper. Res..

[26] Gao W., Fan H., Li W., Wang H. (2021). Crafting the customer experience in omnichannel contexts: the role of channel integration. J. Bus. Res..

[27] Tai F., Wang C., Luo C. (2021). Technology- or human-related service innovation? Enhancing customer satisfaction, delight, and loyalty in the hospitality industry. Service Business.

[28] Quach S., Barari M., Moudrý D.V., Quach K. (2022). Service integration in omnichannel retailing and its impact on customer experience. J. Retailing Consum. Serv..

[29] Barbosa J., Casais B. (2022). The transformative and evolutionary approach of omnichannel in retail companies: insights from multi-case studies in Portugal. Int. J. Retail Distrib. Manag..

[30] Blom A., Lange F., Hess R.L. (2021). Omnichannel promotions and their effect on customer satisfaction. Eur. J. Market..

[31] Cotarelo M., Calderón H., Fayos T. (2021). A further approach in omnichannel LSQ, satisfaction and customer loyalty. Int. J. Retail Distrib. Manag..

[32] Hajdas M., Radomska J., Silva S.C. (2022). The omni-channel approach: a utopia for companies?. J. Retailing Consum. Serv..

[33] Ikpegbu E., Ndinojuo B.-C., Gbeneka E., Diegbegha Y., Onyekasor A. (2017). An assessment of Dano milk radio advertisement on buying behavior of residents of Port Harcourt Local Government, Rivers State, Nigeria. Journal of Marketing and Consumer Research.

[34] Mishra S., Malhotra G., Chatterjee R., Shukla Y. (2023). Consumer retention through phygital experience in omnichannel retailing: role of consumer empowerment and satisfaction. J. Strat. Market..

[35] Moliner M.A., Tortosa-Edo V. (2023). Multirooming: generating e-satisfaction throughout omnichannel consumer journey design and online customer experience. Journal of Research in Interactive Marketing.

[36] Muthaffar A., Vilches-Montero S. (2023). Empowering retailers: a bounded rationality perspective to enhancing omnichannel journey satisfaction. J. Retailing Consum. Serv..

[37] Lehrer C., Trenz M. (2022). Omnichannel business. Electron. Mark..

[38] Taverner C., Trojan L., Simion O., Szkudlarek E. (2021). Design culture in the era of industry 5.0: a review of skills and needs. Cultural Management Science and Education.

[39] Ūsas A., Jasinskas E., Streimikiene D. (2023). The impact of quality of C2C online store on consumer satisfaction: an empirical study in Lithuania. Manag. Market..

[40] Yin C., Chiu H., Hsieh Y., Kuo C. (2022). How to retain customers in omnichannel retailing: considering the roles of brand experience and purchase behavior. J. Retailing Consum. Serv..

[41] Furquim T.S.G., da Veiga C.P., Veiga C.R.P.D., Silva W.V.D. (2023). The different phases of the omnichannel consumer buying journey: a systematic literature review and future research directions. Journal of Theoretical and Applied Electronic Commerce Research.

[42] Huang J., Shi X. (2023). Solving the location problem of front distribution center for omni-channel retailing. Complex & Intelligent Systems.

[43] Shukla A., Tandon J.K. (2019). From brick & mortar channel retailing to omni-channel retailing: exploring shopper's response towards shopping in organized retail industry. Think India Journal.

[44] Lazaris C., Sarantopoulos P., Vrechopoulos A., Doukidis G. (2021). Effects of increased omnichannel integration on customer satisfaction and loyalty intentions. Int. J. Electron. Commer..

[45] Silva S.C., Dias J.C., Braga B. (2023). How footwear companies can use online CX to WOW customers. Int. J. Retail Distrib. Manag..

[46] Tueanrat Y., Papagiannidis S., Alamanos E. (2021). A conceptual framework of the antecedents of customer journey satisfaction in omnichannel retailing. J. Retailing Consum. Serv..

[47] Jasrotia S.S. (2023). The Palgrave Handbook of Interactive Marketing.

[48] Székely S., Csata Z., Cioca L.I., Benedek A. (2020). Industrial marketing 4.0-upgrading the industrial costumers' path to the digital economy. Polish Journal of Management Studies.

[49] Mahadevan K., Joshi S. (2022). Omnichannel retailing: a bibliometric and network visualization analysis. Benchmark Int. J..

[50] Nuanphromsakul K., Szczepańska-Woszczyna K., Kot S., Chaveesuk S., Chaiyasoonthorn W. (2022). Sustainability of rubber farmers' cooperatives: empirical evaluation of determining factors. AGRIS on-line Papers in Economics and Informatics.

[51] Sousa P.R.D., Barbosa M.W., Oliveira L.K.D., Resende P.T.V.D., Rodrigues R.R., Moura M.T., Matoso D. (2021). Challenges, opportunities, and lessons learned: sustainability in Brazilian omnichannel retail. Sustainability.

[52] Yao C., Liao S. (2011). Measuring the antecedent effects of service cognition and internet shopping anxiety on consumer satisfaction with e-tailing service. Manag. Market..

[53] Cattapan T., Pongsakornrungsilp S. (2022). Impact of omnichannel integration on Millennials' purchase intention for fashion retailer. Cogent Business & Management.

[54] Gupta S., Kushwaha P., Badhera U., Chatterjee P., Gonzalez E.D.S. (2022). Identification of benefits, challenges, and pathways in E-commerce industries: an integrated two-phase decision-making model. Sustainable Operations and Computers.

[55] Salvietti G., Ziliani C., Teller C., Ieva M., Ranfagni S. (2022). Omnichannel retailing and post-pandemic recovery: building a research agenda. Int. J. Retail Distrib. Manag..

[56] Silva S.C., Silva F.P., Dias J.C. (2024). Exploring omnichannel strategies: a path to improve customer experiences. Int. J. Retail Distrib. Manag..

[57] Yunita D., Adam M., Wahab Z., Andriana I., Nailis W. (2024).

[58] Orús C., Ibáñez-Sánchez S., Flavián C. (2021). Enhancing the customer experience with virtual and augmented reality: the impact of content and device type. Int. J. Hospit. Manag..

[59] Venkatesh V., Morris M.G., Davis G.B., Davis F.D. (2003). User acceptance of information technology: toward a unified view. MIS Q..

[60] Chao C. (2019). Factors determining the behavioral intention to use mobile learning: an application and extension of the UTAUT model”. Front. Psychol..

[61] Ayaz A., Yanartaş M. (2020). An analysis on the unified theory of acceptance and use of technology theory (UTAUT): acceptance of electronic document management system (EDMS). Computers in Human Behavior Reports.

[62] Mensah I.K., Khan M.K. (2024). Unified Theory of Acceptance and Use of Technology (UTAUT) model: factors influencing mobile banking services' adoption in China. Sage Open.

[63] Batucan G.B., Gonzales G.G., Balbuena M.G.B., Pasaol K.R., Seno D.N., Gonzales R.R. (2022). An extended UTAUT model to explain factors affecting online learning system amidst COVID-19 pandemic: the case of a developing economy. Frontiers in Artificial Intelligence.

[64] Farzin M., Fattahi M. (2022). Investigating the adoption of mobile banking and mobile payment services in developing countries. Reference Module in Social Sciences.

[65] Xue L., Rashid A.M., Ouyang S. (2024).

[66] Chaudhary P., Singh A., Sharma S. (2022). Understanding the antecedents of omni-channel shopping by customers with reference to fashion category: the Indian Millennials' perspective. Young Consum..

[67] Jia X., Pang Y., Huang B., Hou F. (2023). Understanding consumers' continuance intention to watch streams: a value-based continuance intention model. Front. Psychol..

[68] Juaneda-Ayensa E., Mosquera A.M., Sierra Murillo Y.S. (2016). Omnichannel customer behavior: key drivers of technology acceptance and use and their effects on purchase intention. Front. Psychol..

[69] Wolf L., Steul-Fischer M. (2022). Factors of customers' channel choice in an omnichannel environment: a systematic literature review. Management Review Quarterly.

[70] Ahmad F., Mustafa K., Hamid S.A., Khawaja K.F., Zada S., Jamil S., Qaisar M.N., Anwer N. (2022). Online customer experience leads to loyalty via customer engagement: moderating role of value co-creation. Front. Psychol..

[71] Cheah J., Lim X., Ting H., Liu Y., Quach S. (2022). Are privacy concerns still relevant? Revisiting consumer behaviour in omnichannel retailing. J. Retailing Consum. Serv..

[72] de Souza S., Emanoel D., Baldanza R.F. (2018). The e-consumer in light of the perceived value theory: a study on the acceptance of mobile commerce. Base Revista de Administração e Contabilidade da UNISINOS.

[73] Kim D., Kim N., Oh C., Jang J., Nho H., Park S. (2022). Effect of a taekwondo academy's technology-based self-service on perceived value and intention to use continuously in the interaction between humans and technology. Appl. Sci..

[74] Leung K.H., Mo D.Y., Ho G.T., Wu C.H., Huang G.Q. (2020). Modelling near-real-time order arrival demand in e-commerce context: a machine learning predictive methodology. Ind. Manag. Data Syst..

[75] Ylilehto M., Komulainen H., Ulkuniemi P. (2021). The critical factors shaping customer shopping experiences with innovative technologies. Baltic J. Manag..

[76] Chaveesuk S., Khalid B., Chaiyasoonthorn W. (2021). Digital payment system innovations: a marketing perspective on intention and actual use in the retail sector. Innovat. Market..

[77] Ramadan Z., Nsouli N.Z. (2022). Luxury fashion start-up brands' digital strategies with female Gen Y in the Middle East. J. Fash. Mark. Manag.: Int. J..

[78] Cai Y.J., Lo C.K. (2020). Omni-channel management in the new retailing era: a systematic review and future research agenda. Int. J. Prod. Econ..

[79] Rydecki J., Chłąd M. (2023). Sustainability in global supply chains: a consumer-centric perspective. Global Journal of Entrepreneurship and Management.

[80] Khalid B. (2022). Entrepreneurial insight of purchase intention and co-developing behavior of organic food consumption. Polish Journal of Management Studies.

[81] Masorgo N. (2022). The future of omni-channel retail: predictions in the age of amazon, by Lionel Binnie. Transport. J..

[82] Pereira M.M., Frazzon E.M. (2019). Towards a predictive approach for omni-channel retailing supply chains. IFAC-PapersOnLine.

[83] Mrutzek-Hartmann B., Kotzab H., Yumurtacı Hüseyinoğlu I.Ö., Kühling S. (2022). Omni-channel retailing resources and capabilities of SME specialty retailers–insights from Germany and Turkey. Int. J. Retail Distrib. Manag..

[84] Saleh A.A. (2022).

[85] Mirzabeiki V., Saghiri S.S. (2020). From ambition to action: how to achieve integration in omni-channel?. J. Bus. Res..

[86] Zhang M., He X., Qin F., Fu W., He Z. (2019). Service quality measurement for omni-channel retail: scale development and validation. Total Qual. Manag. Bus. Excel..

[87] Chen T.Y., Yeh T.L., Wu H.L., Deng S. (2023). Effect of channel integration quality on consumer responses within omni-channel retailing. Asia Pac. J. Mark. Logist..

[88] Fihartini Y., Helmi R.A., Hassan M., Oesman Y.M. (2021). Perceived health risk, online retail ethics, and consumer behavior within online shopping during the COVID-19 pandemic. Innovat. Market..

[89] Ghani F.A., Rasli M.A.M., Zamri M.A., Salleh N.S.A., Idris R.S.N.R., Razali N.A. (2020). Acceptance of omni-channel retailing among university student: application of UTAUT2 on buying intention. Global Business & Management Research.

[90] Lim X.J., Cheah J.H., Dwivedi Y.K., Richard J.E. (2022). Does retail type matter? Consumer responses to channel integration in omni-channel retailing. J. Retailing Consum. Serv..

[91] Ummah M.H., Azmi N.L., Sharipudin M.S., Fauzi M.S., Abdullah N.A. (2021). The determinants of purchase intention on Omni channel retailing among young Malaysian consumers. Journal of Halal Service Research.

[92] Nanda P., Patnaik S. (2023). A multi-agent coalition-based approach for order fulfilment in e-commerce. Decision Analytics Journal.

[93] Cheung M.L., Pires G.D., Rosenberger P.J., Leung W.K., Salehhuddin Sharipudin M. (2021). The role of consumer-consumer interaction and consumer-brand interaction in driving consumer-brand engagement and behavioral intentions. J. Retailing Consum. Serv..

[94] Jamil K., Dunnan L., Gul R.F., Shehzad M.U., Mustafa Gillani S.H., Awan F.H. (2021). Role of social media marketing activities in influencing customer intentions: a perspective of a new emerging era. Front. Psychol..

[95] Wolfinbarger M., Gilly M.C. (2003). eTailQ: dimensionalizing, measuring and predicting etail quality. J. Retailing.

[96] Neslin S.A., Grewal D., Leghorn R., Shankar V., Teerling M.L., Thomas J.S., Verhoef P.C. (2006). Challenges and opportunities in multichannel customer management. J. Serv. Res..

[97] Hausman A.V., Siekpe J.S. (2009). The effect of web interface features on consumer online purchase intentions. J. Bus. Res..

[98] Lin H.H., Wang Y.S. (2006). An examination of the determinants of customer loyalty in mobile commerce contexts. Inf. Manag..

[99] Porcu L., Del Barrio-García S., Kitchen P.J. (2017). Measuring integrated marketing communication by taking a broad organisational approach: the firm-wide IMC scale. Eur. J. Market..

[100] Horstmann F. (2017). Measuring the shopper's attitude toward the point of sale display: scale development and validation. J. Retailing Consum. Serv..

[101] Lee Y., Choi S., Field J.M. (2020). Development and validation of the pick-up service quality scale of the buy-online-pick-up-in-store service. Operations Management Research.

[102] Dastane O., Goi C.L., Rabbanee F.K. (2023). The development and validation of a scale to measure perceived value of mobile commerce (MVAL-SCALE). J. Retailing Consum. Serv..

[103] Bauer H.H., Falk T., Hammerschmidt M. (2006). eTransQual: a transaction process-based approach for capturing service quality in online shopping. J. Bus. Res..

[104] Angelidou G., Aguaded-Ramirez M.E., Rodriguez-Sabiote C. (2018). Design and validation of a scale measuring attitudes toward refugee children. Sustainability.

[105] Hair J.F., Black W.C., Babin B.J., Anderson R.E., Tatham R.L. (2006).

[106] Kline R.B. (2011).

[107] Becker J.M., Klein K., Wetzels M. (2012). Hierarchical latent variable models in PLS-SEM: guidelines for using reflective-formative type models. Long. Range. Plann..

[108] Fornell C., Larcker D.F. (1981). Evaluating structural equation models with unobservable variables and measurement error. J. Market. Res..

[109] Mofokeng T.E., Tan A.W.K. (2021). The impact of online shopping attributes on customer satisfaction and loyalty: moderating effects of e-commerce experience. Cogent Business & Management.

[110] Davis-Sramek B., Mentzer J.T., Stank T.P. (2008). Creating consumer durable retailer customer loyalty through order fulfillment service operations. J. Oper. Manag..

[111] Rahman S.M., Carlson J., Gudergan S.P., Wetzels M., Grewal D. (2022). Perceived omnichannel customer experience (OCX): concept, measurement, and impact. J. Retailing.

[112] Xuan Q.T., Truong H.T.H., Quang T.V. (2023). The impacts of omnichannel retailing properties on customer experience and brand loyalty: a study in the banking sector. Cogent Business & Management.

[113] Cohen J. (1988).

[114] Boyne G.A. (2002). Public and private management: what's the difference?. J. Manag. Stud..

